# A Biotic Support During Pregnancy to Strengthen the Gastrointestinal Performance in Puppies

**DOI:** 10.3389/fvets.2020.00417

**Published:** 2020-08-04

**Authors:** Monica Melandri, Giulio Guido Aiudi, Michele Caira, Salvatore Alonge

**Affiliations:** ^1^Societá Veterinaria “Il Melograno” Srl, Varese, Italy; ^2^Dipartimento di Medicina Veterinaria, Università degli Studi di Bari Aldo Moro, Bari, Italy

**Keywords:** colostrum, diarrhea, gastroenteritis, prebiotics, probiotics, puppies

## Abstract

Up to 60% of neonates can be affected by gastroenteritis due to specific pathogens or aspecific polymicrobial interactions. The present study evaluated if a dietary supplementation with MOS, FOS, *E. faecium* and *L. acidophilus* in pregnancy may reduce gastroenteritis in puppies. Fifteen Great Danes were divided in 3 groups. The control group (CG) ate a standard diet. In 2 study groups, the diet was supplemented with pre- and probiotics during the last (1WG) and the last 4 pregnancy weeks (4WG). Up to 9 weeks, puppies were checked daily to identify first- or second- presentation gastroenteritis. Data were processed by χ^2^ (*P* < 0.05). First-presentation gastroenteritis was more frequent in CG than in 1WG than in 4WG. Second-presentation gastroenteritis was more frequent in CG than in 1 and 4WG. Puppies from pre- and probiotics supplemented bitches were less prone to gastroenteritis. 1 or 4WG equally reduced second-presentation gastroenteritis in puppies, but 4WG was better than 1WG on first-presentation gastroenteritis. By entero-mammary link, supplemented bitches produced higher immune quality colostrum, thus puppies faced immunitary challenges better; moreover, maternal microbiota, positively altered by supplementation, was transferred to newborns, becoming more resistant to gastroenteritis. This information can be useful in clinical practice with the goal of preventing gastroenteritis in puppies and reducing its prevalence and severity.

## Introduction

Gastroenteritis is one of the most common conditions in canine practice, being particularly frequent among puppies: up to 60% of ill neonates can be affected by diarrhea, caused by either specific pathogens or aspecific polymicrobial interactions (1).

Recently, veterinary researchers focused their attention on the possible effect of gastrointestinal microbiota on canine health and diseases: in adult dogs, pre- and probiotics administered on daily basis are able to modulate the gastrointestinal microbiota, leading to spontaneous recovery from diarrhea, thus avoiding the use of antibiotics and their possible side effects, including the spread of antibiotic resistance (2). Pre- and probiotics represent an effective treatment of gastrointestinal disorders also in young subjects of different species; in German Shepherd and Yorkshire puppies, *Lactobacillus rhamnosus* and *Lactobacillus plantarum* demonstrated a good potential in the prevention of gastrointestinal infections (3); lactic acid bacteria in specific infant formulations successfully prevented diarrhea episodes in children (4–6); in suckling piglets, a probiotic oral treatment with *Enterococcus faecium* proved to be able to mitigate the incidence and severity of diarrhea (7).

Peripartum nutrition is pivotal in the development of the immune system of neonates: pre- and probiotic supplementation of the maternal diet during pregnancy can enhance the immunitary power of colostrum confirming the existence of an enteromammry link (8). The possibility to modulate the composition of the microbiota of both mother and offspring was hypothesized, too, thinking of a microbiota transfer from the mother to the newborns (3).

The present study aimed to evaluate if the maternal dietary supplementation with MOS (mannan-oligosaccharides), FOS (fructo-oligosaccharides), *E. faecium* and *L. acidophilus* during pregnancy could reduce the prevalence of single and multiple clinical episodes of gastroenteritis in the offspring.

## Materials and Methods

### Ethics

The study was performed in accordance with the animal welfare committee ethical guidelines and all procedures were carried out according to the Italian legislation on animal care (DL 116, 27/01/1992) and the European Guidelines on Animal Welfare (Directive 2010/63/EU). Owner's informed consent on the whole procedure was obtained. The present study was based on a retrospective analysis of clinical data gathered during a previous research (9) concerning the effect of maternal diet supplementation with pre- and probiotics on the Ig composition of colostrum and maternal serum, which was approved by the Ethical Committee of Università degli Studi di Bari Aldo Moro (Italy), under protocol CESA-DIMEV Bari n. 20/19.

### Animals

Clinical records of 93 Great Dane puppies were retrospectively evaluated. They were born from bitches enrolled in a previous study concerning the effect of maternal diet supplementation with pre- and probiotics on the immunoglobulin (Ig) composition of colostrum and maternal serum (9).

Fifteen Great Dane bitches (3–6 years; 55–68 kg) were recruited in a FCI kennel, which follows strict guidelines concerning animal health and welfare.

All the animals were housed indoors in identical environmental conditions during the complete assay period since the dog breeder does not sell puppies until they reach the age of ≥75 days. Bitches were regularly vaccinated according to WSAVA 2015 Vaccination Guidelines (10), which means every third year against distemper (CDV), infectious hepatitis (CAV), parvovirosis (CPV2), and parainfluenzavirus (CPiV) (Nobivac CEPPi, MSD Animal Health srl, Milano, Italy) and annually for selected noncore diseases, i.e., leptospirosis and kennel cough (Nobivac L4 and Nobivac KC, MSD Animal Health srl, Milano, Italy). During the month preceding the expected heat, animals were checked for protective antibody titres against CDV, CPV2, and CAV (11) and dewormed with fenbendazole (Panacur Forte, MSD Animal Health srl, Milano, Italy). Each dog underwent a clinical examination, to be proven healthy, including a thorough history evaluation, as well as a female breeding soundness exam (with clinical and ultrasonographic evaluation of the reproductive organs) before the beginning of pregnancy in order to avoid the effect of maternal illness on perinatal health (12, 13).

Bitches and puppies were housed indoors in identical environmental conditions during the whole assay period, with the same estrus and gestation monitoring, parturition, litters, and health management (12, 13).

### Breeding Management

The breeding management from estrus to parturition was similar in all litters. The day of ovulation was identified when the plasma progesterone concentration ranged between 4 and 10 ng/mL (14, 15), as evaluated using an enzyme-linked fluorescent assay (MiniVidas, BioMerieux, Marcy l'Etoile, France). Bitches were mated once 48 h later with males of proven fertility (16, 17).

The day of delivery was estimated from the blood progesterone concentration during estrous and confirmed by fetal biometry (18, 19). Fetal health was assessed by fetal heart rate (20). For all the patients, C-section was planned to protect the mother's and puppies' health, because of previous or expected troubles at parturition (12).

Elective C-section (21) was performed at term and following the clinical appearance of the onset of the first stage of labor, the clinical signs of which were represented by the vaginal elimination of the mucous plug and behavioral alterations (i.e., isolation, restlessness, and lack of appetite) (12).

### Feeding

All the bitches were fed the same dosed commercial diet (Adult Maintenance, Nutrix Più srl, Castelraimondo, Italy) according to metabolic requirements for gestation and lactation, and received water ad libitum. According to the European Pet Food Industry Federation (FEDIAF) 2016 Guidelines, the total daily food intake was calculated basing on maintenance energy requirements (MER, kcal/die). The MER were calculated for each dog, considering the 4 k coefficients, which are represented by breed, attitude, physiologic condition, and health (22).

The 15 bitches involved in the breeding scheme had been previously distributed into one of the 3 study groups according to a randomization list taking into account that each group had to include at least one litter for every season.

The bitches were divided in 3 groups. The control group (CG) received only a standardized commercial diet. The diet was supplemented with a mix of pre- and probiotics (Florentero, Candioli SRL, Beinasco, Italy) during the last (1WG), and the last 4 (4WG) weeks of pregnancy.

The supplement is composed as follows: prebiotics: FOS 40%+ MOS 4,05%; probiotics 8.86 × 109 CFU/g of supplement: E.faecium DSM10663/NCIMB104154b17072, 80 × 108 CFU/g + L. acidophilus CECT 4529 4b1715 8,58 × 109 CFU/g. This supplement is formulated in tablets, 1.2 g each. Consequently, each tablet contains: FOS 480 mg, MOS 48.6 mg, E. faecium 3.36 × 108 CFU, L. acidophilus 1.03 × 1010 CFU. Following the manifacturer's instruction, animals received one tablet every 10 kg of bodyweight.

This specific supplement was chosen in the present study because its ingredients are currently commonly used in veterinary clinical practice. Moreover, they were individually tested in previous studies on similar topics, related to gestation, lactation, and weaning, in the canine species as well as in other animals (3, 7–9, 23, 24).

### Litters Management

At whelping, neonates were clinically examined and the neonatal bodyweight was measured before the first suckling. For all the puppies, the first suckling took place immediately after this first clinical evaluation, at the end of the C-section. No remarkable differences could be described in the 9-week-breeding management that could artificially enhance a different neonatal/puppies survival/health in any of the groups. All the litters were kept in optimal standardized environmental conditions and puppies received maternal milk, only, until weaning from 25 days of age with the same dosed commercial diet (Cuccioli Svezzamento, Nutrix Più srl, Castelraimondo, Italy). Puppies were dewormed at 20 days with pyrantel pamoate (Nemex, Zoetis, Roma, Italy) and at 40 days with fenbendazole (Panacur, MSD Animal Health srl, Milano, Italy) and first vaccinated at 57 days of age (Nobivac CEPPi, MSD Animal Health srl, Milano, Italy) (11). During the 9-week-procedure follow-up, puppies were provided access to socialization and exercise according to their age in an open area adjacent to the house.

### Procedure

In the selected kennel, all animals are daily checked by a veterinarian, who reports their clinical data on a litter sheet. In the present study, information from the litter sheet was retrospectively analyzed.

Up to 9 weeks of age, puppies were daily checked individually to promptly identify any case of gastroenteritis, diagnosed when loose or watery stools were reported, with or without vomiting or fever (3), differentiating cases of first or second presentation. The veterinarian entrusted to evaluate feces was blind to the maternal diet during gestation.

### Statistical Analysis

Data obtained from clinical evaluations were reported on an Excel 2010 Office file.

For both first- and second-presentation gastroenteritis, the ratio between the number of affected subjects and the total number of puppies born in each study group was calculated. The ratio was also expressed as percentage. These data were statistically processed by the chi-squared test.

Litters and puppies numbers, male/female ratio, litter size, maternal age, and parity in the 3 study groups were statistically compared by ANOVA to check the absence of epidemiological differences between groups.

Results were considered significant for *P* <0.05. The statistical analysis was performed with the online tools VassarStats: Website for Statistical Computation (http://vassarstats.net, Vassar College, New York, NY, USA) and Social Science Statistics (https://www.socscistatistics.com, Jeremy Stangroom, USA).

## Results

All parturitions were at term, by elective C-section, with fully developed (25) alive puppies without complications; birth weight was within the normal ranges reported for the breed (26). Thirty-one, 30 and 32 puppies were born in CG, 1, and 4WG, respectively. No mortality was observed during the whole essay period.

No statistically significant differences among the 3 study groups were found concerning epidemiological parameters, such as litters and puppies number (Table 1), male/female ratio, litter size, maternal age, and parity, that could have artificially biased the results.

**Table 1:** Number of puppies in each litter in the 3 study groups.

**Litters**	**1**	**2**	**3**	**4**	**5**	***N*^**°**^ total**	**Mean ± SD**
Control group (CG)	6	7	12	4	2	31	6.2 ± 3.77*
1 week (1WG)	1	10	13	2	4	30	6 ± 5.24*
4 weeks (4WG)	7	5	9	4	7	32	6.4 ± 1.95*

Equal superscripts (*) denote the absence of any statistical difference within the mean number of puppies in the 3 study groups (p > 0.05).

First-presentation gastroenteritis was significantly more frequent in CG (23/31; 74.2%) than in 1WG (12/30; 40.0%). It was observed to a significantly less extent in 4WG (5/32; 15.6%) than in CG and 1WG (Table 2).

**Table 2:** Intestinal episodes in the 3 study groups.

**Intestinal episodes**	**1**	**2**
	***N* (%)**	***N* (%)**
Control group (CG)	23/31 (74.2)*	10/31 (32.3)*
1 week (1WG)	12/30 (40.0)**	0/30 (0)**
4 weeks (4WG)	5/32 (15.6)***	1/32 (3.1)**

Different superscripts (*, **, ***) denote statistical differences within columns (p <0.05).

Second-presentation gastroenteritis was significantly more frequent in CG (10/31; 32.3%) than in 1WG (0/30; 0%), and 4WG (1/32; 3.1%) (Table 2).

## Discussion

Pre- and probiotics are known to offer a non-pharmacological alternative to manage several gastoenteric disorders in dogs, both adults and puppies, through a direct daily alimentary administration, improving animals microbiota, and gut health (2, 3). They act through different systems: direct effect on pathogens (competitive exclusion); enhancement of adaptive and innate immune response, including phagocytosis (immunomodulation); increased digestive efficiency (23).

The results of the present study demonstrate that puppies born from pre- and probiotics supplemented mothers are less prone to both first- and second-presentation gastroenteritis. One or 4 weeks of supplementation during pregnancy equally reduced repeated gastroenteritis in puppies, but 4WG was more effective than 1WG to reduce the prevalence of gastroenteritis since the first presentation. Two possible explanations can be given: first, thanks to the enteromammy link, supplemented bitches produce a colostrum with a higher immune quality (8) and, after a correct colostrum assumption, their puppies are more capable to face immunitary challenges; second, the maternal microbiota is positively altered by the supplementation and is then transferred to the newborns, who become more resistant to gastroenteritis.

Referring to colostrum, previous results demonstrated that bitches receiving a diet supplemented with pre- and probiotics during gestation produce a colostrum with higher immune quality (8, 9). In particular, a supplementation period of 4 weeks leads to a significant increase in the IgG and IgM content, while a 2-week-supplemetation already influences the concentration of IgA positively (9). The immunitary power of colostrum, enhanced by the maternal diet supplementation with pre- and probiotics, effectively reaches puppies only when its intake occurs before the intestinal barrier closure in puppies, that is to say within the first 12 h after birth (27). This time was complied with for all the puppies enrolled in the present study, whose first suckling took place immediately at the end of the C-section. The IgG (27) and IgA (28) are described as the main source of immune protection for puppies in the very first weeks of life. The IgG are mainly involved in systemic protection, but, after colostrum absorption, a reverse secretion into the intestinal lumen is reported, where they probably contribute to reduce the replication of pathogens (29). Conversely, IgA are less involved in systemic immunity, while they play a crucial role on mucous surfaces (30). In fact, even after the gut closure, IgA are able to adhere to the gastrointestinal surface, creating a protective coating of the epithelial layer, facing enteropathogens within the intestinal lumen (31), actively binding to micro-organisms and enterotoxins to prevent their adherence and penetration into the gastrointestinal wall (32, 33). Moreover, IgA are likely specific against environmental agents (29). The mother already lives in the same environment where puppies will grow, thus she produces IgA specific for those microbial agents existing where puppies will live their first weeks of life. The age at which puppies start producing local IgA on their own still remains to be defined (30) but seems to take place later in life (34). Thus, neonates are more probably protected against environmental agents, potentially causing gastroenteritis, thanks to IgA of maternal origin, that pass through the colostrum (8).

Referring to microbiota, the possibility was formulated of a microbial transfer from the mother to the fetus and newborn (3). Thus, a dietary-based modulation of the maternal microbiota could indirectly be transferred to the puppies. The choice of the right probiotics administered to the bitch turns out to be fundamental for the health of her offspring, including the prevention of gastroenteritis episodes. *Enterococcus faecium* was the main bacterium represented in the supplementation used in the present study. It is a lactic acid bacterium, normal inhabitant of the gut flora in animals (35) and humans (36), with inhibitory power against relevant enteropathogens, i.e., enterotoxigenic *Escherichia coli, Salmonella Spp., Shigella* Spp. and *Clostridium* Spp. (37). Thus, *Enterococcus faecium* is commonly added to functional food, as an anti-diarrhoeal agent (23). It was reported that *Enterococcus fecium* plays a relevant role in a preferential IgA switch of mucosally primed B cells associated with an increase in mucosal IgA respose due to the specific homing of the B cells producing IgA in the gut (23, 38, 39). It was also stated that the combined use of prebiotics and probiotics could have a cumulative effect on the regulation of gut immunity, including an increase in IgA production (24). This action of *Enterococcus faecium*, coupled to the synergistic effect of prebiotics and *Lactobacillus acidophilus*, could take place both in supplemented bitches, who would then produce a colostrum richer in IgA, and in their puppies, whose B cells, that are mucosally primed by *Enterococcus faecium*, would produce increased levels of IgA.

Literature reports that stress and dietary changes can affect the gastrointestinal microbiota in dogs (23). Neonatal growth and weaning can be considered representative of both these phenomena. In these special phases of the puppies' life, a favorable microbiota composition (23), also of maternal origin, could represent a key point to improve both present and future health conditions, particularly referring to the development of the immune system.

Further studies would be advisable to evaluate possible differences in the composition of intestinal microbiota and fecal immunoglobulins of puppies with and without gastroenteritis episodes, born from pre- and probiotic-supplemented and non-supplemented mothers.

It was demonstrated that pre- and probiotics administered to the mother during pregnancy increase colostrum quality with higher immunogobulin concentrations (8). Since colostrum plays a pivotal role in the digestive immunity and in the development of the gastrointestinal microbiota, of Peyer's patches, and of the digestive epithelium (31), puppies receiving a colostrum with higher immunitary properties will undergo a better stimulation of the intestinal immune system, essential to formulate proper immune responses and to limit infections, inflammations, and allergies (40, 41). A longer supplementation of the maternal diet with pre- and probiotics, as in the 4WG group, seems to give the best clinical results on the offspring in terms of gastroenteritis prevention.

## Data Availability Statement

The data that support the findings of this study are available from the corresponding author, upon reasonable request.

## Ethics Statement

The animal study was reviewed and approved by the Animal Welfare Committee Ethical Guidelines and all procedures were carried out according to the Italian Legislation on Animal Care (DL 116, 27/01/1992) and the European Guidelines on Animal Welfare (Directive 2010/63/EU). Owner's informed consent on the whole procedure was obtained. The present study was based on a retrospective analysis of clinical data gathered during a previous research (9) concerning the effect of maternal diet supplementation with pre- and probiotics on the Ig composition of colostrum and maternal serum, which was approved by the Ethical Committee of Università degli Studi di Bari Aldo Moro (Italy), under protocol CESA-DIMEV Bari n. 20/19. Written informed consent was obtained from the owners for the participation of their animals in this study.

## Author Contributions

MM and SA: conceptualization, investigation, data curation, formal analysis, methodology, visualization, and writing–original draft preparation. GA: validation. GA and MC: funding. MM, GA, MC, and SA: writing–review and editing.

## Conflict of Interest

MM and SA are employed by the company Societá Veterinaria “Il Melograno” Srl. The remaining authors declare that the research was conducted in the absence of any commercial or financial relationships that could be construed as a potential conflict of interest.
